# Physical activity level of female and male adult cats before and after running wheel habituation

**DOI:** 10.1017/jns.2017.19

**Published:** 2017-05-15

**Authors:** Katelyn B. Detweiler, Samona Rawal, Kelly S. Swanson, Maria R. C. de Godoy

**Affiliations:** Department of Animal Sciences, University of Illinois, Urbana, IL 61801, USA

**Keywords:** Exercise, Feline nutrition, Obesity, Running wheels, BW, body weight, FAA, food anticipatory activity

## Abstract

The objective of the present study was to evaluate whether access to a running wheel increases voluntary physical activity in adult female and male domestic cats. Eight neutered domestic shorthair male cats (mean age 8·6 (sd 0·05) years) and eleven intact domestic shorthair female cats (mean age 3·3 (sd 0·14) years) were group housed for 22 h daily and individually housed during the feeding period. Voluntary physical activity was measured using accelerometers. Experimental design consisted of 1 week of baseline physical activity measurement, followed by 3 weeks of wheel habituation, and 1 week of physical activity measurement post-wheel habituation. Female cat voluntary physical activity levels increased (*P* < 0·05) post-habituation during the dark period, resulting in an altered (*P* < 0·05) light:dark activity ratio, whereas male cat voluntary physical activity levels remained unchanged post-habituation. Food anticipatory activity did not differ pre- and post-habituation. However, it corresponded to a numerically greater proportion of daily physical activity for males (17·5 %) *v*. females (12 %). In general, female cats were more active than male cats. Habituation to a running wheel appears to be an effective method to increase voluntary physical activity of younger female cats. Thus, running wheels might be a potential strategy in the prevention or management of feline obesity.

Obesity is one of the most common diseases present in companion animals today. Recent studies suggest that approximately 58 % of adult cats in the USA are either overweight or obese^(^^1^^)^. Additionally, decreased physical activity has been identified as a major risk factor for the development of feline obesity and diabetes^(^^2^^)^. Thus, different strategies to increase physical activity in domestic cats need to be explored in conjunction with dietary management to decrease the occurrence of obesity. One possible strategy to increase physical activity would be the implementation of a running wheel designed for domestic cats.

Several running wheels designed for domestic cats are available commercially, but their effectiveness in increasing voluntary physical activity is unknown. Thus, the objective of this study was to evaluate whether access to a running wheel increases voluntary physical activity in adult female and male domestic cats. We hypothesised that cats habituated to using the running wheel would have greater voluntary physical activity than prior to wheel access and habituation.

## Materials and methods

### Animals and diets

All animal care procedures were approved by the University of Illinois Institutional Animal Care and Use Committee. Eight neutered domestic shorthair male cats (mean age: 8·6 (sd 0·05) years) and eleven intact domestic shorthair female cats (mean age 3·3 (sd 0·14) years) were used in this experiment. The cats were group-housed for 22 h daily and individually housed in individual stainless-steel cages (1·02 × 0·76 × 0·71 m^3^) during the feeding period (08.00–10.00 hours). The cats were housed into two rooms based on sex. The rooms were environmentally controlled with a 16 h light–8 h dark cycle and had similar enrichment furniture (i.e. scratching poles, climbing trees) and various toys.

Cats were fed an experimental dry kibble diet at amounts to maintain body weight (BW) and body condition score throughout the study. The diet contained 5·1 kcal (21·3 kJ)/g, 37·2 % crude protein and 16·5 % acid-hydrolysed fat on a DM basis. Food refusals were collected and weighed daily. Cats were weighed weekly. Water was available *ad libitum* throughout the experiment.

### Experimental design

Baseline voluntary physical activity of female and male cats was determined 7 d prior to the experiment to establish baseline level of exercise so each cat could be used as its own control. Directly following the baseline measurement, a 21-d period of wheel habituation occurred so that the cats were trained to use the running wheel. During this period, research personnel worked with the cats individually for 10 min/d, 5 d/week, to encourage them to use the wheels. Cats were habituated at the same time every section. Toys or a laser pointer were used to encourage the use of the wheel. After the habituation period, there was a 7-d period of voluntary physical activity measurement post-habituation with no human interference (excluding feeding time).

### Voluntary physical activity assessment

Voluntary physical activity was evaluated using Actical activity monitors (Mini Mitter). The monitors contain omnidirectional sensors capable of accurately incorporating both intensity and duration of movement. The use of activity monitors for physical activity assessment has been validated^(^^3^^)^ and allowed an objective measure of the physical activity of the cats without human interference. The Actical software analysed the data compiled by the monitor and converted it into arbitrary numbers referred to as ‘activity counts’. Physical activity was expressed as activity counts per epoch (epoch length = 15 s). Values represent the mean epoch activity count over a 7-d period, during the selected hours (light hours, dark hours, and average daily activity). To control for variability, the cats wore the same monitor among experimental periods. Actical monitors were activated at 08.00 hours on day 1 and turned off at the same time on day 7.

### Food anticipatory activity

Food anticipatory activity (FAA) was calculated for all cats prior to and after wheel habituation. This measurement corresponded to the 2 h of voluntary physical activity that preceded the scheduled feeding time (06.00–08.00 hours) divided by the total daily voluntary physical activity and expressed as a percentage^(^^3^^)^. FAA was calculated from day 2 to day 6.

### Statistical analyses

All data were analysed using the Mixed procedure of a commercial software package (SAS version 9.4; SAS Institute, Inc.). Data normality was analysed using PROC UNIVARIATE. Cat was included in the model as a random effect. The fixed effects of period, sex and their interaction were tested in the model. Treatment least squares means were compared with each other, and the Tukey adjustment was used to control for experiment-wise error. A probability of *P* ≤ 0·05 was considered significant.

## Results

### Voluntary physical activity

An increase (*P* < 0·05) in female cat voluntary physical activity levels post-habituation was observed during the dark period, resulting in an altered (*P* < 0·05) light:dark activity ratio. Male cat voluntary physical activity levels remained unchanged post-habituation. A sex effect (*P* = 0·008) was observed for voluntary physical activity during the light period, with male cats being 42 % less active than female cats (Table 1). Significant main effects of sex (*P* < 0·0001) and treatment (*P* = 0·008) were observed for BW, despite cats been fed to maintain their baseline weight. Male cats in this study had a considerably larger body size (sexual dimorphism) when compared with the female cats, and thus this outcome was somewhat expected.

**Table 1.** Comparison of voluntary physical activity pre- and post-habituation between male (*n* 8) and female (*n* 11) cats (Mean values and pooled standard errors)

	Treatment				Statistics			
	Female		Male					
Items	Pre-habituation	Post-habituation	Pre-habituation	Post-habituation	sem	Treatment	Sex	Interaction
Daily physical activity	24·9^x^	31·4^y^	13·7^x^	13·6^x^	3·015	0·07	0·002	0·063
Light physical activity	26·5	26·7	15·4	15·2	2·892	0·999	0·008	0·904
Dark physical activity	21·6^a^	36·1^b^	10·1^a^	10·2^a^	3·911	0·018	0·001	0·02
Light:dark ratio	1·4^a^	0·9^b^	1·6^a^	1·5^a^	0·138	0·006	0·022	0·014
Body weight (kg)	3·7	3·6	5·1	5·0	0·129	0·008	<0·001	0·855

a,b Mean values within a row with unlike superscript letters denote a significant interaction (*P* < 0·05).

x,y Mean values within a row with unlike superscript letters denote a trend to a significant interaction (*P* < 0·10).

### Food anticipatory activity

**Fig. 1.**
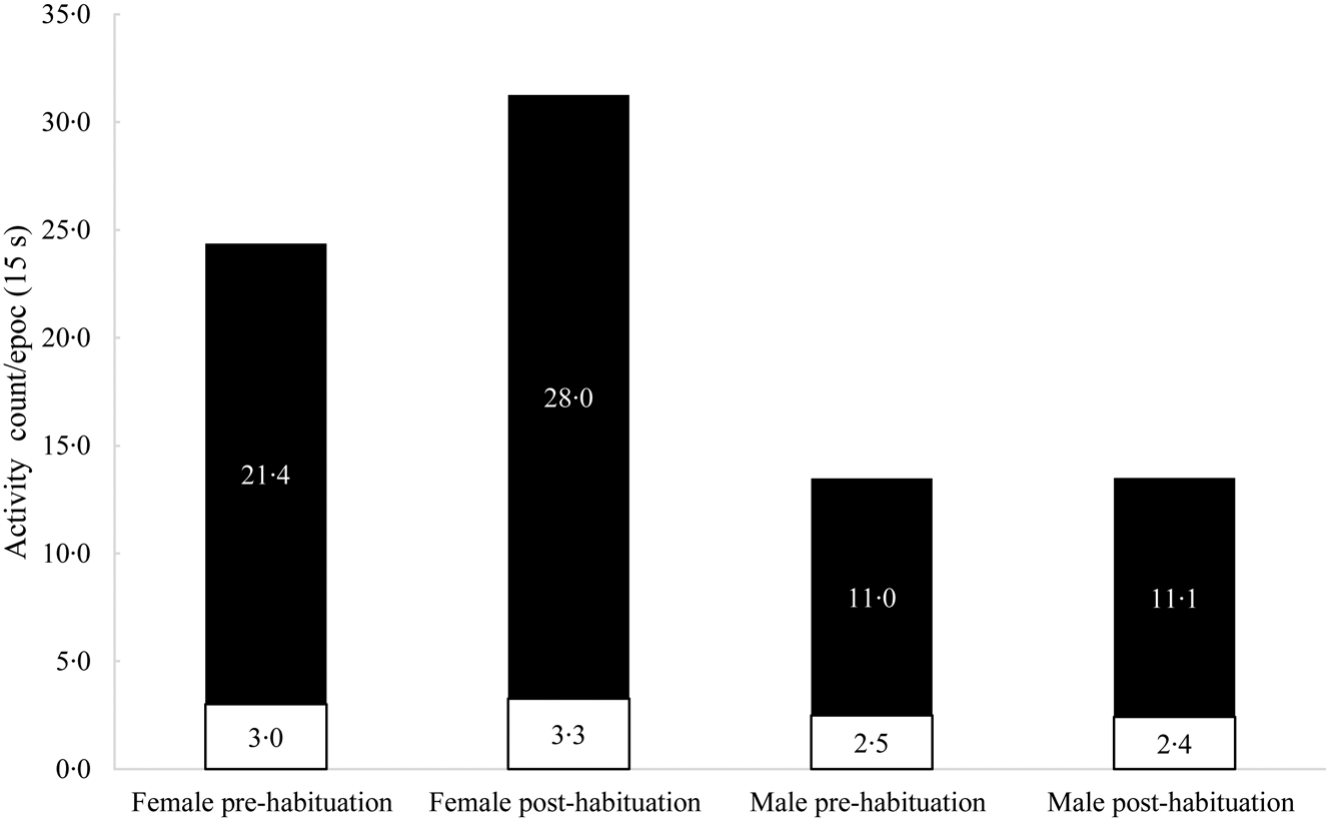
Average total daily voluntary physical activity (■) and food anticipatory activity (FAA; □) for male (*n* 8) and female (*n* 11) cats before and after habituation to a running wheel. The average total daily voluntary physical activity, including FAA, is represented by the entire bar while the white portion represents the average FAA. The white and black portions of each column are summative to reflect total daily voluntary physical activity.

Despite female cats being more active than male cats post-habituation, FAA did not differ (*P* > 0·05) in female and male cats pre- and post-habituation. However, FAA corresponded to a numerically greater proportion of daily physical activity for male (17·5 %) *v*. female (12 %) cats (Fig. 1).

## Discussion

Decreased physical activity has been shown to contribute to feline obesity and diabetes in domestic cats^(^^4^^)^. The purpose of this study was to assess the effectiveness of a running wheel in increasing physical activity in adult domestic cats. Our hypothesis was that habituation to a running wheel would increase physical activity in cats. To our knowledge, no studies have been done that evaluated the effectiveness of a running wheel on physical activity in domestic cats. However, previous research from our laboratory has investigated the effects of castration, environmental, and/or dietary manipulation on voluntary physical activity in cats^(^^3^^,^^5^^–^^7^^)^.

In support of our hypothesis, habituation to the running wheel increased the voluntary physical activity of female cats, which also had a greater daily voluntary physical activity and physical activity during the dark period when compared with the male cats. The increased physical activity during the dark cycle resulted in an altered light:dark activity ratio of female cats. In contrast to our hypothesis, however, average voluntary daily physical activity of male cats did not change after wheel habituation, nor did their physical activity during the light and dark cycles. This could suggest that sex, age and castration, or a combination of these factors, could influence the voluntary physical activity of adult cats. Corroborating this proposition, Belsito *et al.*^(^^7^^)^ reported that spaying drastically decreased (50 %) the voluntary physical activity of young adult female cats (average 1·5 years). In addition, in that study similar total daily physical activity counts (30 (se 1·82) counts) were reported in female cats prior to spaying intervention.

De Godoy *et al*.^(^^5^^)^ reported numerically lower average daily activity for female cats fed dry food multiple meals per d *v*. one meal per d (18·3 *v*. 15·8 (se 1·72) counts per epoch, respectively), in contrast with our findings. However, the present study followed a similar activity pattern (data not shown) to previous studies in our laboratory^(^^5^^,^^6^^)^. The female cat post-habituation physical activity counts were within ranges reported in a previous study that evaluated the effect of feeding frequency on voluntary physical activity of cats^(^^6^^)^.

In this study, the pre- and post-habituation FAA was similar in male and female cats. However, because the males had less activity counts overall, their FAA contributed to a greater proportion of the daily physical activity (17·5 %) *v*. the female cats (12 %). De Godoy *et al*.^(^^5^^)^ discussed that a lower food intake and metabolic energy requirement per kg BW of neutered cats could lead to a greater feeling of hunger and search for food, leading to an increase in FAA behaviour. Similarly, in this study, the female cats had a greater food intake of 14·5 g/kg BW (45 % above) compared with the male neutered cats, which consumed 10 g/kg BW. A sharp decline in the voluntary physical activity of female and male cats was observed post-feeding (data not shown). This finding is also in agreement with previous studies from our laboratory^(^^3^^,^^5^^)^.

### Conclusions

In conclusion, according to the data presented herein, the use of running wheels might be an effective method to increase voluntary physical activity of younger female cats, but not older male cats. Therefore, a running wheel might be a potential strategy in the management and prevention of feline obesity. However, further studies are warranted as this study had a few limitations that could have confounded the results, as the female cats were younger and intact whereas the male cats were older and neutered. Thus, evaluation of the effects of age, sex and neutering, as well as their potential interactions, on the voluntary physical activity of domestic cats is needed as these factors could not be distinguished in this study. Another limitation was that the time spent by the cats in the running wheel pre- and post-habituation was not measured. Nonetheless, because there were no changes in the daily care, environment and housing of the cats, the increase in voluntary physical activity observed in this study is probably due to the cats having access and being habituated to the running wheel.
